# GATA Binding Protein 4 Regulates Tooth Root Dentin Development *via* FBP1

**DOI:** 10.7150/ijbs.36567

**Published:** 2020-01-01

**Authors:** Yuxin Zhang, Mengru Fang, Zhiwen Yang, Wenhao Qin, Shuyu Guo, Junqing Ma, Wenjing Chen

**Affiliations:** Jiangsu Key Laboratory of Oral Diseases, Nanjing Medical University, Nanjing, China.

**Keywords:** knockout mice, neural crest, multipotent stem cells, dentinogenesis, odontoblasts, gluconeogenesis

## Abstract

Tooth development is a complex process that is regulated precisely by several signalling pathways and transcription factors. GATA-binding protein 4 (GATA4) is a DNA binding transcription factor, and our previous study showed that GATA4 is a novel regulator of root development. However, it remains unclear whether GATA4 is necessary for odontoblast differentiation and dentin formation. Here, we evaluated the phenotypic changes of *Wnt1-Cre;GATA4^fl/fl^* mice. The mutant mice showed defective dentin and short root deformity. The odontoblasts lost polarity instead of exhibiting a shorter height and flattened morphology. Moreover, the expression of several molecules, such as DSPP, COL-1, DCN, and PCNA, were downregulated during mutant tooth development.* In vivo*, we injected lentivirus to overexpress GATA4 in mice root. The dentin formation and the expression of odonto/osteogenic markers (DSPP, COL-1, DCN) were enhanced in the GATA4 overexpression group. During the* in vitro* study, the ability of proliferation, migration and odonto/osteogenic differentiation was declined by GATA4 knockdown approach in human dental pulp stem cells (DPSCs). The expression of odonto/osteogenic markers (DSPP, BMP4, RUNX2, OSX, OPN, OCN) was reduced in the shGATA4 group, while overexpressing GATA4 in DPSCs promoted mineralization. Furthermore, an immunoprecipitation-mass spectrometry procedure was used to confirm the interaction between GATA4 and Fructose-1, 6-bisphosphatase 1 (FBP1). We used gain and lose-of-function to delineated the role of GATA4 in regulating FBP1 expression. Knocking down GATA4 in DPSCs resulted in decreased glucose consumption and lactate production. We used small hairpin RNA targeting FBP1 to reduce the expression of FBP1 in DPSCs, which significantly increased glucose consumption and lactate production. Together, the results suggested that GATA4 is important for root formation and odontoblast polarity, as it promotes the growth and differentiation of dental mesenchymal cells around the root and affects the glucose metabolism of DPSCs *via* the negative regulation of FBP1.

## Introduction

Teeth are ectodermal organs, and their development involves the sequential steps of reciprocal epithelial-mesenchymal interactions 1. During tooth development, the stages of crown and root formation are distinct. Tooth roots develop following crown formation, a process that is initiated by the formation of the Hertwig's epithelial root sheath (HERS) 2, which subsequently induces the internal dental mesenchymal cells to differentiate into matrix-producing odontoblasts to form dentin. Dentin, a major mineralized component of teeth, is primarily composed of type I collagen. The functional odontoblast has a columnar shape, and its major function is to secrete matrix and regulate matrix mineralization. The polarization of odontoblasts is a key factor in regulating extracellular matrix mineralization 3.

GATA binding protein 4 (GATA4) is a zinc finger-containing transcription factor 4 that is well known for its central role in cardiac development 5. Several studies have reported that GATA4 is an important factor in osteoblasts differentiation 6,7. Our previous study showed that GATA4 is a novel regulator of root development, and GATA4 protein expression was found in the majority of cells in the dental mesenchyme, odontoblasts, and ameloblasts during tooth development at embryonic day 14.5 (E14.5), E15.5, and E17.5, as well as postnatal day 1 (P1) and P14 8,9. Previous analyses of mouse models that conditionally knockout GATA4 expression in neural crest cell (NCC) derivatives revealed that* Wnt1-Cre; GATA4^fl/fl^* mice have short root deformity. However, the role of GATA4 in postnatal odontoblast development and dentin formation has not been precisely studied. This finding aroused our interest in studying the role of GATA4 in tooth morphogenesis and dentin formation, which may facilitate the development of new therapies for developmental deformities of the root.

In mammalian cells, the catabolic glycolysis/oxidative phosphorylation pathway and the anabolic gluconeogenesis pathway are two major ways used to maintain glucose homeostasis 10. Dental pulp stem cells play an important role in the formation of teeth, with an odonto/osteogenic differentiation potential. Wang et al. 11 reported that glycolysis increased when DPSCs initiate differentiation. Fructose-1, 6-bisphosphatase 1 (FBP1) is a key regulatory enzyme during the process of gluconeogenesis 12, but the mechanism by which FBP1 influences tooth development and the relationship between GATA4 and FBP1 remain unknown.

In the present study, we investigated the function of GATA4 in regulating the growth, differentiation and glucose metabolism of dental mesenchymal cells during tooth root development, as well as the role of FBP1 in dentinogenesis. Moreover, our work showed that FBP1 mediates these processes under the regulation of GATA4.

## Materials and Methods

### Animals

Mice were obtained from the Model Animal Research Center (MARC) at Nanjing University. The Institutional Animal Care and Use Committee at the Nanjing Medical University approved all experimental procedures. To specifically remove GATA4 in NCC-derived dental mesenchymal stem cells, we crossed *GATA4^fl/fl^* females (C57/BL6) with *Wnt1-Cre; GATA4^fl/-^* males.

### Micro-CT analysis

Genetically modified mice were sacrificed at various time points and fixed in freshly prepared 4% paraformaldehyde (PFA) overnight at 4°C. The slice thickness for micro-CT scans was 18 μm at 50 kV and 456 μA 13. Images were reconstructed and analysed using NRecon v1.6 and CTAn v1.13.8.1 software (Bruker, Germany).

### Histological analysis

*Wnt1-Cre; GATA4^fl/fl^* and *GATA4^fl/fl^* mice were harvested at P1, 7, 14 and 21. Skulls were carefully dissected and fixed in freshly prepared 4% paraformaldehyde overnight at 4°C. Then, tissues were decalcified in 10% DEPC-treated EDTA (pH 7.4) for 1-4 weeks depending on the age of the sample. Decalcified tissues were dehydrated and paraffin embedded, and 5-µm thick sections prepared. Haematoxylin and eosin (H&E) staining 14 and immunohistochemical examination 15 using standard procedures were performed to examine the phenotypic changes and molecular expression. For immunohistochemical examination, polyclonal rabbit anti-DSPP (1:200), anti-COL-1 (1:200), anti-DCN (a proteoglycan, decorin) (1:100), anti-GATA4 (1:200) and anti-proliferating cell nuclear antigen (PCNA) (1:200) were used as primary antibodies (The detailed information of each antibody was showed in Table S1.). Finally, immune complexes were visualized using a diaminobenzidine (DAB) kit.

### Lentivirus injection

The P7 mice (C57/BL6) were anesthetized through the inhalation of ether. Mice in the experimental group received 5 μL concentrated lentiviral supernatant (GATA4 OE; 1 × 10^9^ TU/mL) injected under the buccal periosteum of the left mandibular first molar using a microsyringe, whereas control group mice received the same dose of lentivirus delivering a nonspecific sequence (Ctrl OE). For the long-term gene silencing experiments, two injections were added every three days till alveolar bones were too hard to perform injection. Mice were harvested for histological analysis at P17 16. Lentivirus to overexpress GATA4 (GATA4 OE) in mouse and blank lentivirus (Ctrl OE) were purchased from GenePharma (Shanghai, China).

### Isolation and culture of human dental pulp stem cells

Human dental pulp stem cells (DPSCs) isolated from normal human third molars without caries were acquired from patients (age: 16-20 years) treated at the Oral Surgery Department of the Jiangsu Provincial Stomatological Hospital (Nanjing, China). All experiments were performed using a protocol approved by the Ethical Committee at the Stomatological School of Nanjing Medical University (Nanjing, China). Dental pulp tissues were gently separated, minced and digested 17. Single-cell suspensions were obtained and cultured in α-MEM medium 18 at 37°C in a 5% CO_2_ incubator. Culture media were refreshed every 3 days. DPSCs at passage 3-5 were used for cell experiments.

### Cell transfection

Recombinant lentivirus of shRNA target GATA4 (shGATA4; 5′-GAATAAATCTAAGACACCA-3′), control lentivirus (shCTRL; 5′-TTCTCCGAACGTGTCACGT-3′), lentivirus to overexpress GATA4 (pcDNA-GATA4) and blank lentivirus were purchased from GenePharma (Shanghai, China). Efficient shRNA targeting FBP1 was used in this study. The antisense sequence 19 against FBP1 is 5**′**-AACATGTTCATAACCAGGTCG-3**′**. DPSCs were seeded in 6-well culture plates and subsequently infected with the lentivirus (MOI=50) at 60-70% confluence in the presence of 5 μg/mL polybrene 20.

### Flow cytometric (FCM) analysis

DPSCs were used to evaluate stem cell surface markers by flow cytometry. The dental pulp stem cells (1 × 10^6^) were harvested in phosphate-buffered saline (PBS), washed, and fixed with 4% paraformaldehyde (PFA) for 30 min. The fixed cells were then permeabilized and incubated with the antibodies CD14 PE-CY7 (cat. 557742, BD Biosciences, San Jose, USA), CD45 APC (cat. 555485, BD Biosciences, San Jose, USA), CD44-PE (cat. 550989, BD Biosciences, San Jose, USA) and CD90-FITC (cat. 561969, BD Biosciences, San Jose, USA) for 1 hour at 4°C, and the IgG isotype antibodies were used as negative controls for each experiment. The expression profiles were examined using an LSR II flow cytometer system (BD Biosciences) 21. At least 50,000 cells were used for fluorescence-activated cell sorting analysis.

### Cell migration assay using transwell system

Cell migration was assessed using a two-chamber Transwell system (8 mm pore size and 6.5 mm diameter). DPSCs (5 × 10^3^ cells/mL) suspended in 100 μL of serum free α -MEM were planted in the top chamber of the Transwell, and 500 μL of the eluate prepared with serum-free α-MEM was added to the lower chambers. After incubation at 37 °C in 5% CO2 for different time points, filters were removed with sterile tweezers and cells that did not migrate through the filter were gently wiped with a cotton swab. Migrating cells beneath the filter were fixed with 4% paraformaldehyde for 15 min and stained with crystal violet for 20 min. The filters were observed using an inverted microscope (Leica). The numbers of the migrating cells in each well were counted in 6 random microscopic fields per filter at 200× magnification. The experiments were performed in triplicate independently.

### Wound scratch assay

DPSCs transfected with GATA4 lentivirus were seeded on to 6-well plates at a density of 1.5×10^5^ cells/well. When cells grew to 95%-100%, a pipette tip was used to inflict a wound. Debris and floating cells were removed by washing the cells once with 1 mL of the growth medium. Wound closure was measured at different time points (12 and 24 h) using a Leica DMIRE2 microscope in phase contrast mode and Leica FW4000 software (Cambridge, UK) 22.

### Cell counting kit-8 assay

DPSCs transfected with GATA4 lentivirus were seeded on to 96-well plates at a density of 1000 cells/well and cultured in α-MEM with 10% FBS. The cells infected with control lentivirus served as control groups. At specific time points (0 h, 24 h, 48 h, 72 h, 96 h), the cultures were supplemented with cell counting kit-8 (CCK-8) solution (Dojindo, Japan) and incubated for 1h at 37°C. Cell proliferation was measured at a wavelength of 450 nm by a microplate reader 23.

### Alkaline phosphatase activity assay and alizarin red staining

DPSCs were cultured in mineralization-inducing media for 7 days and stained with a BCIP/NBT Alkaline Phosphatase Colour Development Kit (Beyotime Institute of Biotechnology, Shanghai, China) 24. Cells were cultured in mineralization-inducing medium for 2 weeks and stained with 2% Alizarin red (Beyotime Institute of Biotechnology, Shanghai, China). Alizarin red was destained using 10% cetylpyridinium chloride (CPC) in 10 mM sodium phosphate for 30 min 25. The concentration was evaluated by measuring the absorbance at 405 nm with a microplate reader.

### Western blotting

Details of the western blot protocol are described elsewhere 26. Briefly, total protein was collected, boiled, loaded onto a 10% SDS-PAGE gel for separation, and transferred onto polyvinylidene fluoride (PVDF) membranes. After blocking with 5% bovine serum albumin (BSA) for 2 hours, the membrane was incubated overnight at 4°C with primary antibodies against GATA4 (1:1000), dentin sialophosphoprotein (DSPP) (1:500), runt-related transcription factor 2 (RUNX2) (1:500), osterix (OSX) (1:500), osteopontin (OPN) (1:500), osteocalcin (OCN) (1:500), BMP4 (1:500), FBP1 (1:500), β-actin (1:1000) and GAPDH (1:500) (The detailed information of each antibody was showed in Table S1.). Then, membranes were incubated with secondary antibodies at room temperature for 1 hour, rinsed with Tris buffered saline (with Tween-20) thrice, and visualized by enhanced chemiluminescence. Semi-quantitative measurements were performed using Image J software (National Institutes of Health, USA).

### Quantitative reverse transcription polymerase chain reaction for messenger RNA analysis

Total cell RNA was isolated using TRIzol reagent (Invitrogen) according to the manufacturer's protocol 27. mRNA was converted to complementary deoxyribonucleic acid (cDNA) using the HiScript® Q RT SuperMix for qPCR (Vazyme, Nanjing, China). The gene expression level was analysed by quantitative reverse transcription PCR (qRT-PCR) using the ABI-7300 Real-Time PCR System (Applied Biosystems, CA, USA). The primers used are listed below (forward/reverse): RUNX2 (5′-AGTTCCCAAGCATTTCATC-3′/5′-GGCAGGTAGGTGTGGTAGT-3′); OSX (5′-CTACCCATCTGACTTTGCTC-3′/5′-CACTATTTCCCACTGCCTT-3′); OPN (5′-CTCCAATCGTCCCTACAGTCG-3′/5′-CCAAGCTATCACCTCGGCC-3′); OCN (5′-CACACTCCTCGCCCTATT-3′/5′-GGTCTCTTCACTACCTCGCT-3′); BSP (5′-GCTGATGAACGCCTACTGC-3′/5′-AAACCTCGATGGTGTCGC-3′); DSPP (5′-CCATTCCAGTTCCTCAAA-3′/5′-GCCTTCCTCTATCACCTTC-3′); GAPDH (5′-TGAACCATGAGAAGTATGACAACA-3′/5′-TCTTCTGGGTGGCAGTG-3′).

### Co-immunoprecipitation and mass spectrometry

Dental pulp stem cells were cultured in 10-cm dishes to 95% confluency. Cell lysates were collected by scraping cells in cell lysis buffer. After brief sonication, cell lysates were centrifuged, and supernatants were transferred to a fresh 1.5 mL tube. Antibodies were then added to cell lysates and incubated on a rotator in cold room for 2 hours. Prewashed Dynabeads protein G were added to the lysate-antibody mix, incubated for 2 hours in a cold room, and then used to pull down interacting proteins 28. The beads were then washed 4-5 times with cell lysis buffer. The proteins were eluted with SDS sample buffer and heated at 95°C for 5 min. The samples were then run on the Bolt 4-12% Bis-Tris Plus Gel system (Life Technologies) and stained using Bio-Safe Coomassie Stain. Gel lanes were excised into individual fractions. Bands fractions were then further reduced into cubes of 1-2 mm^3^, destained, washed, dried and extracted. Each fraction was reconstituted in 0.1% formic acid and analysed using online liquid chromatography on a nanoAcquity-UPLC coupled to a Thermo LTQ Orbitrap Velos mass-spectrometry.

### Glycolysis analyses

Glucose uptake and lactate production were measured using commercial kits (Nanjing Jiancheng Bioengineering Institute, China) following the standard protocol. The glucose and lactate concentrations were measured according to the OD values at 505 nm and 530 nm 29, respectively, using a microplate reader (Molecular Devices).

### Statistical analyses

All experiments in this study were performed thrice to test the reliability of the results, and important findings are shown. Experimental values are given as the mean ± SD. The significance of differences between the control and treatments was evaluated by Student's t-test (SPSS 19.0). *P* < 0.05 was considered significant.

## Results

### Thin dentin and short roots in molars of *Wnt1-Cre; GATA4^fl/fl^* mice

**Figure 1:**
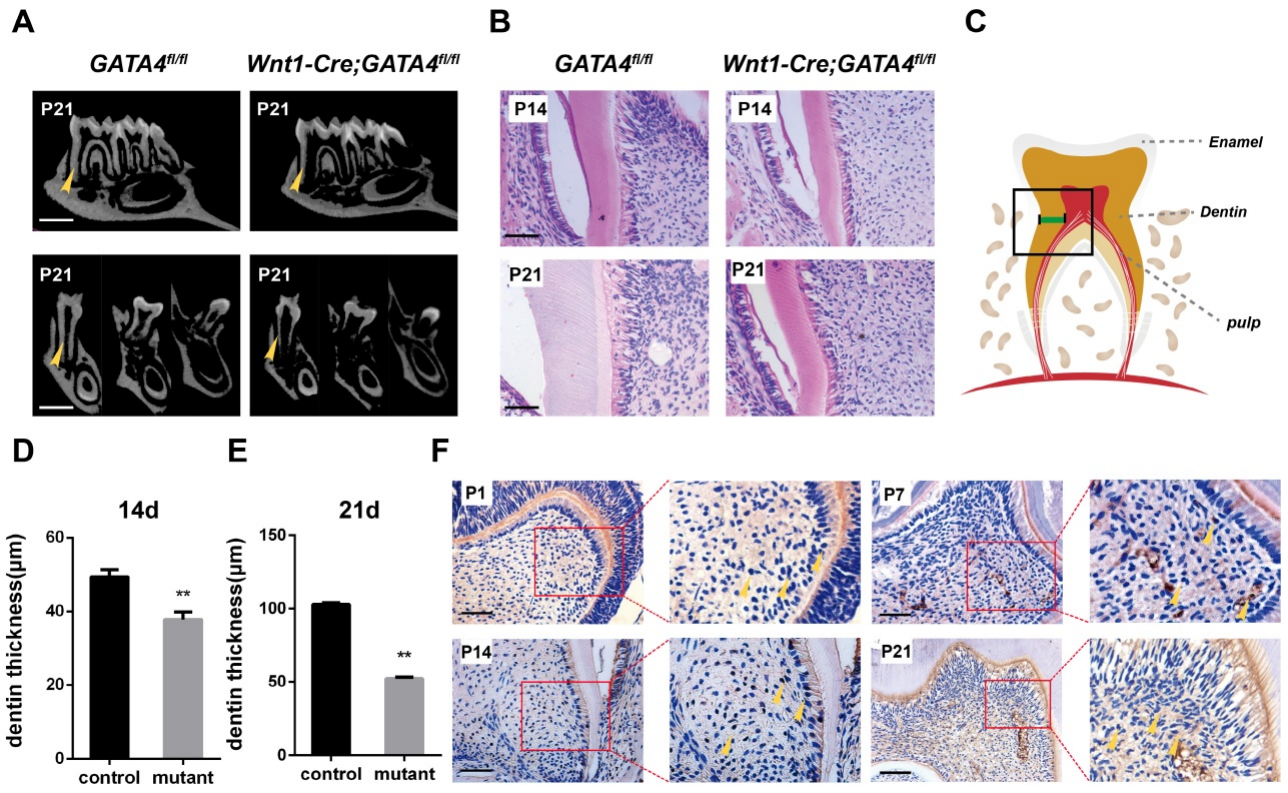
**Tooth root dentin abnormalities in *Wnt1-Cre;GATA4^fl/fl^* mice.** (**A**) Micro-CT showing short roots and dentin defects in the mandibular molars of *Wnt1-Cre;GATA4^fl/fl^* mice at postnatal day 21 (P21) (Bar: 300 μm). (**B**) H&E-stained sections of the mandibular first molars showed that root dentin thickness was decreased in *Wnt1-Cre;GATA4^fl/fl^* mice at P14 and P21 (Bar: 50 μm). (**C**) The thickness of dentin was measured. Quantitative assessment of the molar root dentin thickness at P14 (**D**) and P21 (**E**). (**F**) Expression pattern of GATA4 during tooth development at P1, P7, P14 (Bar: 50 μm) and P21 (Bar: 100 μm), Yellow arrow indicates the expression of GATA4. Data expressed as the mean ± standard deviation, n = 3. ^**^P < 0.01.

To specifically remove GATA4 in dental mesenchymal cells, we crossed a floxed GATA4 allele with a *Wnt1-Cre* driver. Micro-CT analysis of the molars revealed that root length and root dentin thickness was decreased in *Wnt1-Cre; GATA4^fl/fl^* mutants compared with control mice at postnatal day 21 (P21) (Fig. 1A), whereas no differences in crown height and thickness were noted. These findings were also verified in the H&E-stained sections of the mandibular first molars at P14 and P21 (Fig. 1B-E). A previous study showed that GATA4 protein expression was located in the majority of cells in the dental mesenchyme, odontoblasts and ameloblasts at E14.5, E15.5, E17.5, P1 and P14. To further detect the expression and location of GATA4, immunohistochemical analysis was used in the sections of mouse first mandibular molars at P1, P7, P14 and P21 (Fig. 1F). During dentin development, GATA4 was not only highly expressed in odontoblasts but also strongly located in a large number of dental pulp cells (DPCs) in mice. Collectively, these results confirmed GATA4 play an important role in tooth morphogenesis and dentin formation.

### Effect of GATA4 on odontoblasts polarization, cell proliferation and secretion of root dentin matrix

**Figure 2:**
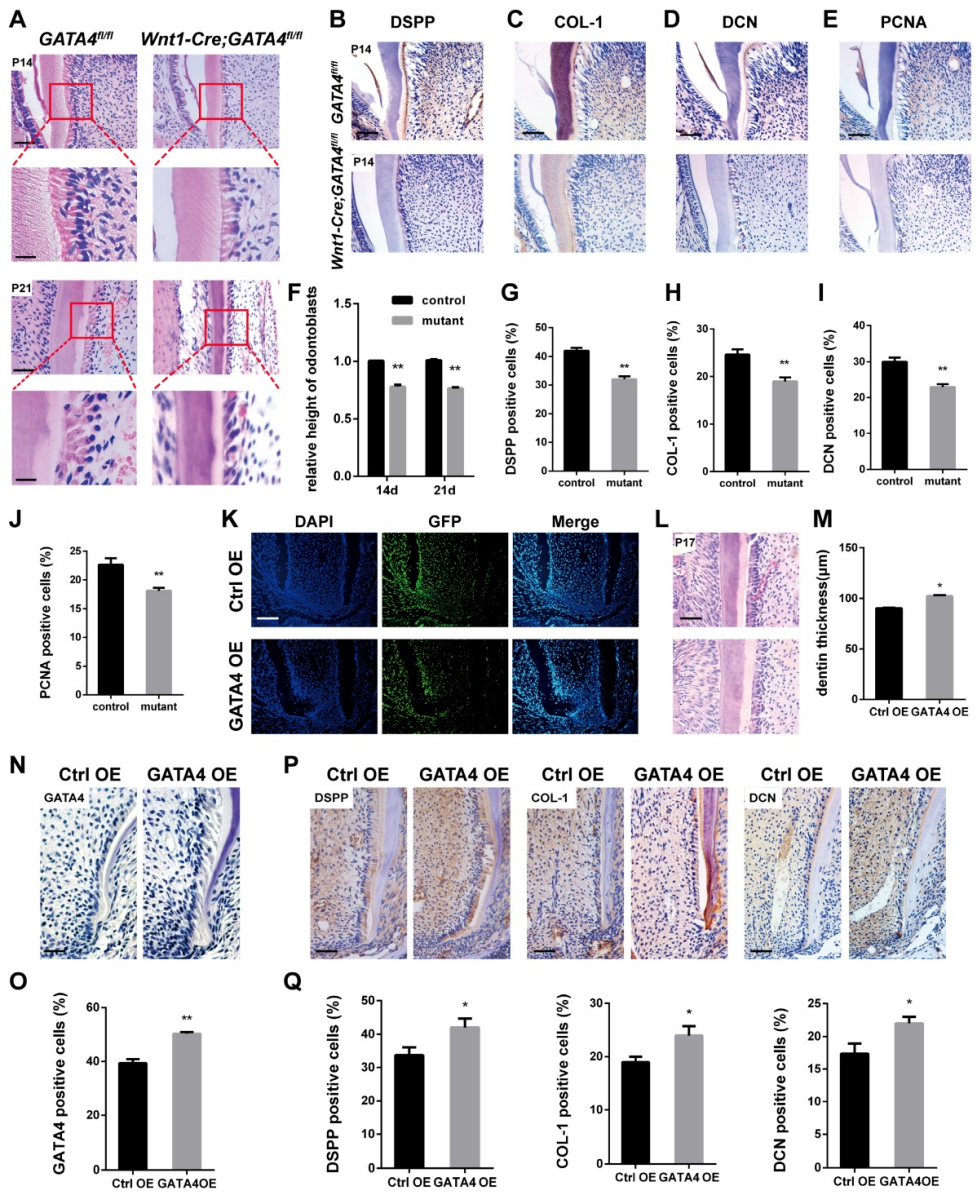
**Effect of GATA4 on odontoblasts polarization, cell proliferation and secretion of root dentin matrix.** (**A**) H&E-stained sections of the mandibular first molars showed that the odontoblasts have shorter height and flattened morphology in *Wnt1-Cre;GATA4^fl/fl^* mice at P14 and P21 (Bar: 50 μm). (**B-E**) Immunohistochemistry staining images showing expression levels of DSPP (B), COL-1 (C), DCN (D), and PCNA (E) in root of *Wnt1-Cre;GATA4^fl/fl^* mice at P14. (**F**) The height of odontoblasts was measured. Quantitative assessment of the molar root odontoblasts height from (A) at P14 and P21. (**G-J**) Percentages of DSPP (G), COL-1 (H), DCN (I), and PCNA-positive (J) cells in the control and mutant groups were calculated. (**K**) Double-labeled fluorescent immunostaining of DAPI-stained cell nuclei (blue), GFP (green), and merged images in tooth root at P17 after the injetion *in vivo* (Bar: 50 μm). (**L**) H&E-stained sections of the mandibular first molars showed that root dentin thickness was increased in GATA4 OE group mice at P17 (Bar: 50 μm). (**M**) Quantitative assessment of the molar root dentin thickness at P17. (**N**) Expression of GATA4 after lentivirus injected at P17 (Bar: 100 μm). (**O**) Percentages of GATA4-positive cells in the two groups were calculated. (**P**) Immunohistochemistry staining images showing expression levels of DSPP, COL-1, and DCN in root of mice at P17. (**Q**) Percentages of DSPP, COL-1, and DCN-positive cells in the two groups were calculated. Data expressed as the mean ± standard deviation, n = 3. ^*^P < 0.05, ^**^P < 0.01.

Well differentiated odontoblasts consistently exhibit a tall columnar shape 3, whereas H&E-stained sections of* Wnt1-Cre;GATA4^fl/fl^* mice teeth showed that the odontoblasts have shorter height and flattened morphology, implying the loss of odontoblast polarity by ablation of GATA4 in odontoblasts (Fig. 2A, F). Immunohistochemistry was performed to further assess the molecular changes. DSPP, COL-1 and DCN were reduced in mutant odontoblasts and dentin (Fig. 2B-D, G-I). We therefore examined whether impeded root growth in the *Wnt1-Cre; GATA4^fl/fl^* mutants is proceeded by defective cell proliferation in the odontoblasts and DPCs of the first molar. We used PCNA immunohistochemistry to label proliferating cells (Fig. 2E, J). In the *Wnt1-Cre; GATA4^fl/fl^* mutants, PCNA-labelled cells were decreased in the odontoblasts and DPCs. To further examine the function of GATA4 in odontoblasts and dentin, we built an animal model. Lentivirus that overexpress GATA4 (GATA4 OE) and the control (Ctrl OE) were injected under the buccal periosteum of the left mandibular first molar using a microsyringe. The selected expression vectors contained a GFP tag; thus, cells infected with the lentivirus would express GFP. Indeed, strong GFP expression was seen in experimental and control mice (Fig. 2K). Then, in order to visualize and verify the expression of GATA4 *in vivo*, immunohistochemistry of sections of alveolar bone using the rabbit polyclonal anti-GATA4 antibody was performed. As expected, more GATA4-labeled cells were detected in experimental mice at P17 (Fig. 2N, O). H&E-stained sections of the mandibular first molars showed that root dentin thickness was increased in experimental mice compared with control mice at P17 (Fig. 2L, M). Moreover, the expression of DSPP, COL-1 and DCN were increased in experimental mice odontoblasts and dentin (Fig. 2P, Q). Collectively, these results showed that GATA4 promotes polarization in odontoblasts, cell proliferation and secretion of root dentin matrix.

### GATA4 expression during odonto/osteogenic differentiation of DPSCs and efficiency of GATA4 knockdown

**Figure 3:**
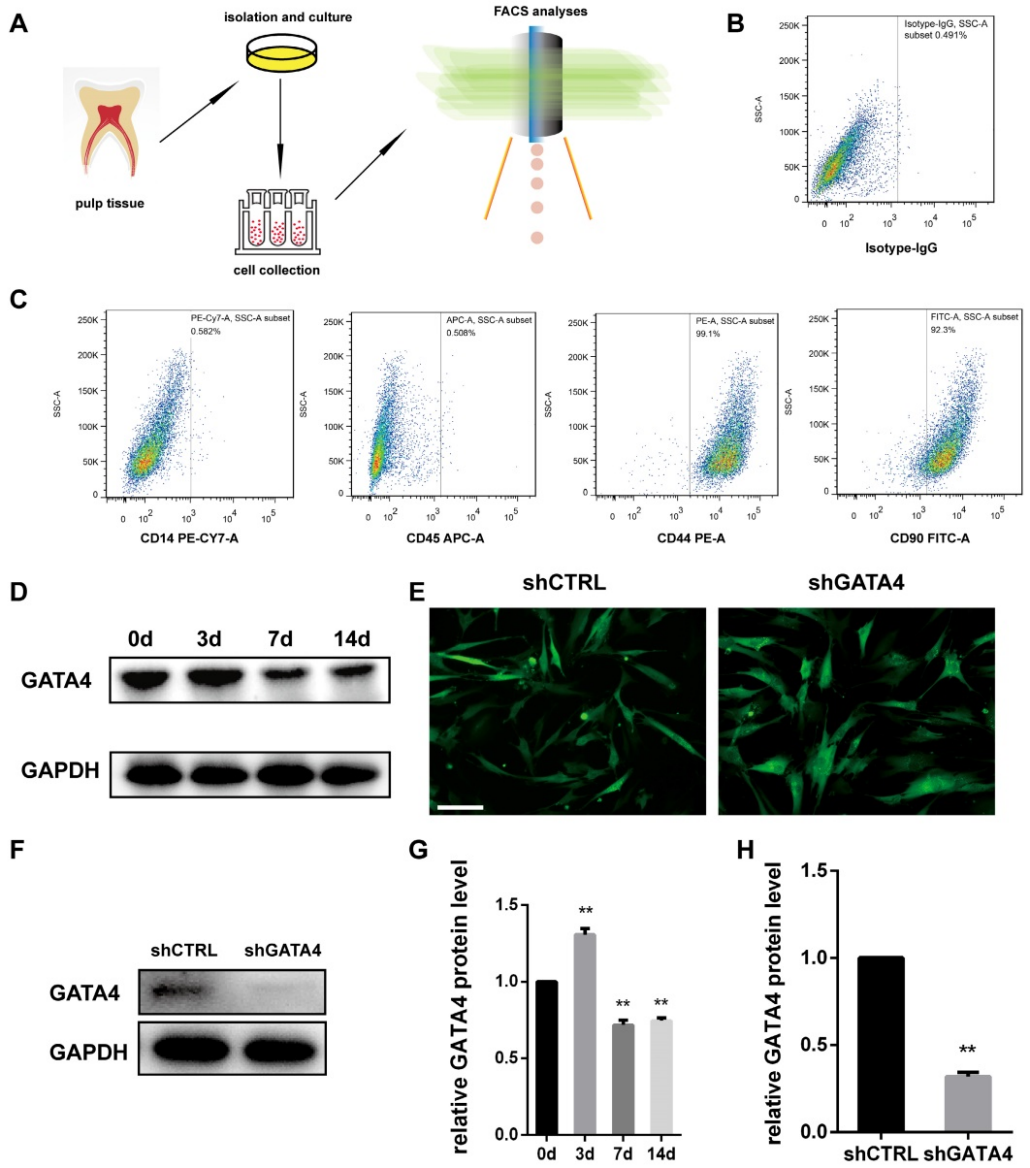
**Characterization of DPSCs and expression of GATA4 in DPSCs.** (**A**) Flow chart explaining cells isolation, culture and collection for FCM analyses. (**B, C**) Flow cytometry demonstrated that DPSCs expressed mesenchymal markers (CD44 and CD90) at a high level and generated the hematopoietic makers (CD14 and CD45) at a low level. (**D**) After mineralization for 3, 7, and 14 days, GATA4 protein expression was assessed at the indicated time points by western blotting. (**E**) DPSCs infected with lentivirus as assessed by fluorescence microscopy (Bar: 50 μm). (**F**) Efficiency of GATA4 knockdown after infection with lentivirus was analysed by western blotting. (**G**) Quantitative analysis of western blotting bands from (D) is shown as the ratio of GATA4 to GAPDH. (**H**) Quantitative analysis of western blotting bands from (F) is shown as the ratio of GATA4 to GAPDH. Data expressed as the mean ± standard deviation, n = 3. ^**^P < 0.01.

Dental pulp stem cells (DPSCs) exhibit a typical fibroblast-like morphology and appeared microscopically as elongated and spindle-shaped cells 30. To investigate the effect of GATA4 during odonto/osteogenic differentiation of DPSCs, we collected fresh human third molars without caries, and DPSCs were isolated, cultured and identified (Fig. 3A-C). Followed it, we examined GATA4 protein expression during odonto/osteogenic differentiation of DPSCs. Western blotting showed that GATA4 expressed at a high level the third day (Fig. 3D, G). To further examine the function of GATA4 in DPSCs, we used lentivirus that expresses specific *Gata4* shRNA to knock down GATA4 in DPSCs. After DPSCs were transfected by lentiviral particles (MOI 50) for 72 hours, we observed green fluorescence in both shCTRL and shGATA4 groups (Fig. 3E). Western blotting analysis confirmed that GATA4 expression in the shGATA4 transfected cells was markedly decreased compared with the shCTRL group (Fig. 3F, H). Collectively, these data showed that GATA4 has an important role during odonto/osteogenic differentiation of DPSCs.

### Knockdown of GATA4 expression impairs migration, proliferation and odonto/osteogenic differentiation of DPSCs

**Figure 4:**
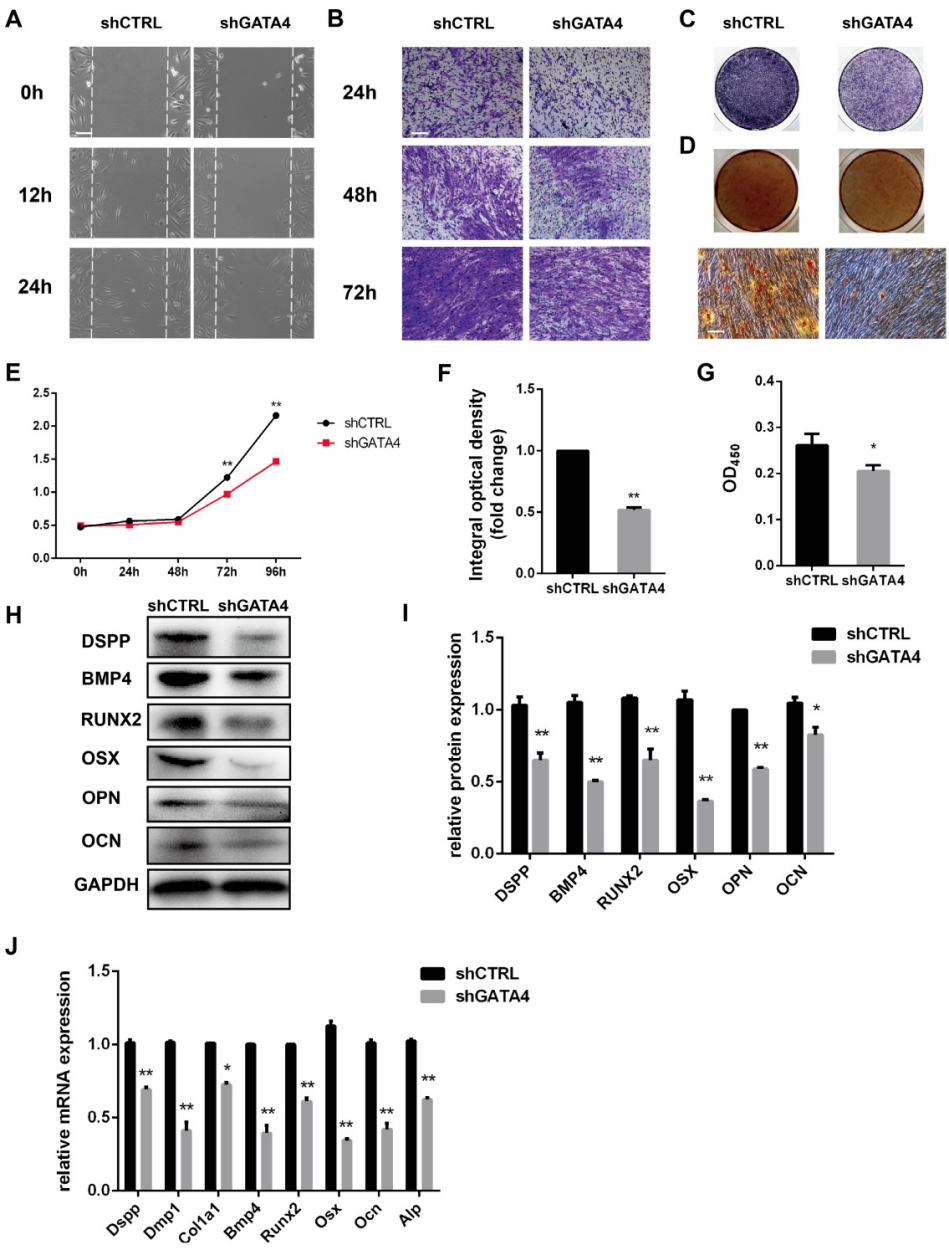
** Effect of GATA4 on migration, proliferation and odonto/osteogenic differentiation of DPSCs.** (**A**) Effect of GATA4 knockdown on cell migration was assessed by wound scratch assays (Bar: 100 μm). (**B**) Effect of GATA4 knockdown on cell migration was assessed by transwell assay (Bar: 100 μm). (**C**) ALP staining observed after 7 days of mineralization (Bar: 100 μm). (**D**) After mineralization for 14 days, alizarin red staining was performed and observed with an image scanner (upper) and under a microscope (lower) (Bar: 100 μm). (**E**) The CCK8 assay was used to analyse the proliferation of DPSCs after infection with GATA4 lentivirus. (**F**) Quantitative assessment of ALP-positive areas. (**G**) Semi-quantitative estimation of calcium. (**H**) Expression levels of odonto/osteogenic-related genes (DSPP, BMP4, RUNX2, OSX, OPN, and OCN) were assessed by western blotting. (**I**) Quantitative analysis of western blotting bands from (H). (**J**) Expressions of odonto/osteogenic markers (Dspp, Dmp1, Col1a1, Bmp4, Runx2, Osx, Ocn, and Alp) were assessed by qRT-PCR. (Bar: 100 μm). Data expressed as the mean ± standard deviation; n = 3. ^*^P < 0.05, ^**^P < 0.01.

To determine whether GATA4 could regulate the migratory ability of DPSCs, wound healing and transwell migration assays were performed (Fig. 4A, B). GATA4 knockdown attenuated migratory ability compared with control groups. The proliferation rates of DPSCs were analysed in response to knockdown of GATA4 expression using CCK8 assays (Fig. 4E). As shown in Fig. 4E, the proliferation rates of the shGATA4 group decreased after 3 and 4 days of culture compared with the control group. ALP staining revealed significant decreases in ALP-positive areas in the shGATA4 group compared with the control group (Fig. 4C, F). Moreover, the results of the alizarin red staining assay were consistent with the ALP assay. After osteogenic induction for 14 days, the shGATA4 group contained a smaller number of mineralization nodules and had lower calcium concentrations than the control group (Fig. 4D, G). The expression of the odonto/osteogenic markers was detected using qRT-PCR and western blotting. Western blotting assays revealed a significant decrease in the expression of odonto/osteogenic markers (DSPP, BMP4, RUNX2, OSX, OPN, OCN) in the shGATA4 group (Fig. 4H, I). Similarly, the expression of odonto/osteogenic markers (Dspp, Dmp1, Col1a1, Bmp4, Runx2, Osx, Ocn, and Alp) at the mRNA level was downregulated in the shGATA4 group (Fig. 4J). Collectively, these results suggested that GATA4 enhance cells migration, proliferation and odonto/osteogenic differentiation of DPSCs.

### Effect of GATA4 overexpression on odonto/osteogenic differentiation of DPSCs

**Figure 5:**
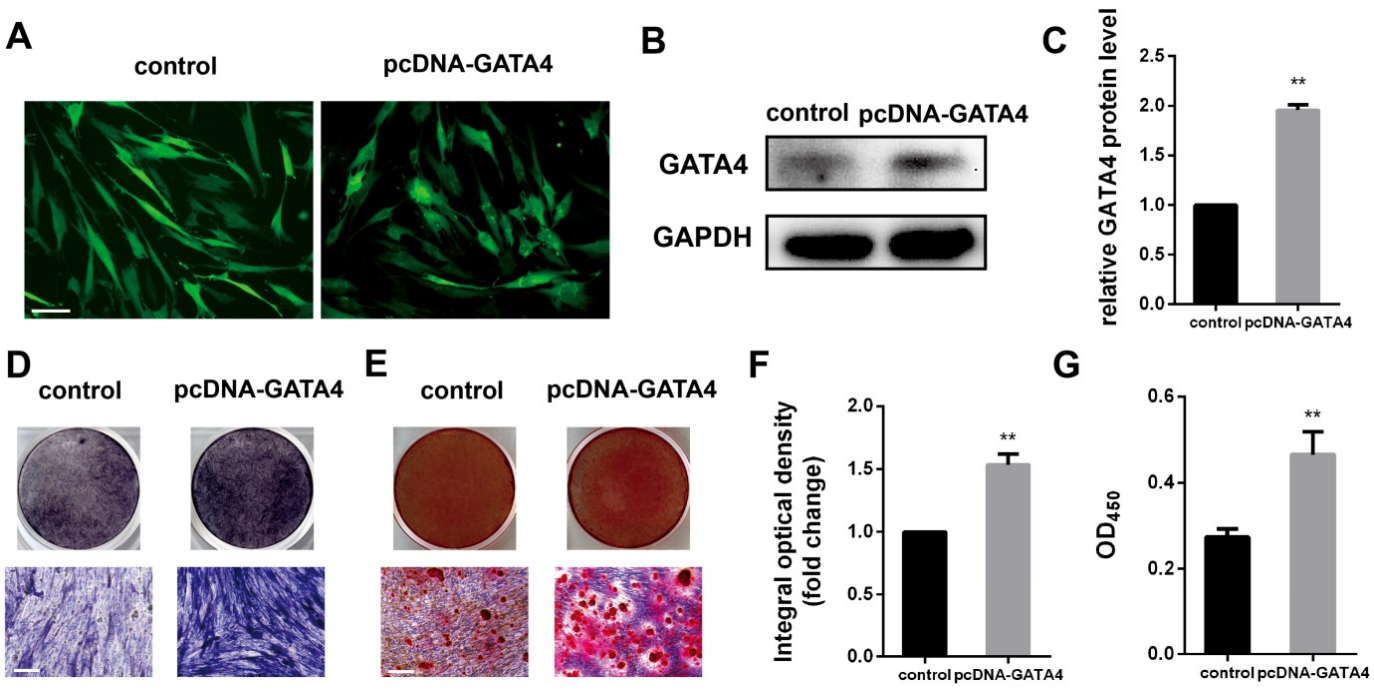
** Overexpression of GATA4 in DPSCs increased the odonto/osteogenic ability.** (**A**) DPSCs infected with lentivirus control and pcDNA-GATA4 were observed under a fluorescence microscope (Bar: 50 μm). (**B**) Protein expression of GATA4 in the DPSCs was tested by western blotting after overexpression of GATA4. (**C**) Quantitative analysis of western blotting bands from (B). (**D**) ALP staining was observed after 7 days of mineralization. (**E**) ARS staining was performed 14 days after mineralization (Bar: 100 μm). (**F**) Quantitative assessment of ALP-positive areas after 7 days of osteogenic induction. (**G**) Semi-quantitative estimation of calcium. Data expressed as the mean ± standard deviation; n = 3. ^**^P < 0.01.

DPSCs infected with lentivirus expressing pcDNA-GATA4 were able to overexpress GATA4 by up to 80% (Fig. 5A-C). Compared with control group, cells in the pcDNA-GATA4 group underwent odonto/osteogenic differentiation and mineralization as demonstrated by increased ALP staining (Fig. 5D, F), ARS staining and calcium concentrations (Fig. 5E, G). Collectively, these data indicate that GATA4 is essential in DPSCs odonto/osteogenic differentiation and mineralization.

### GATA4 knockdown affects FBP1 expression

**Figure 6:**
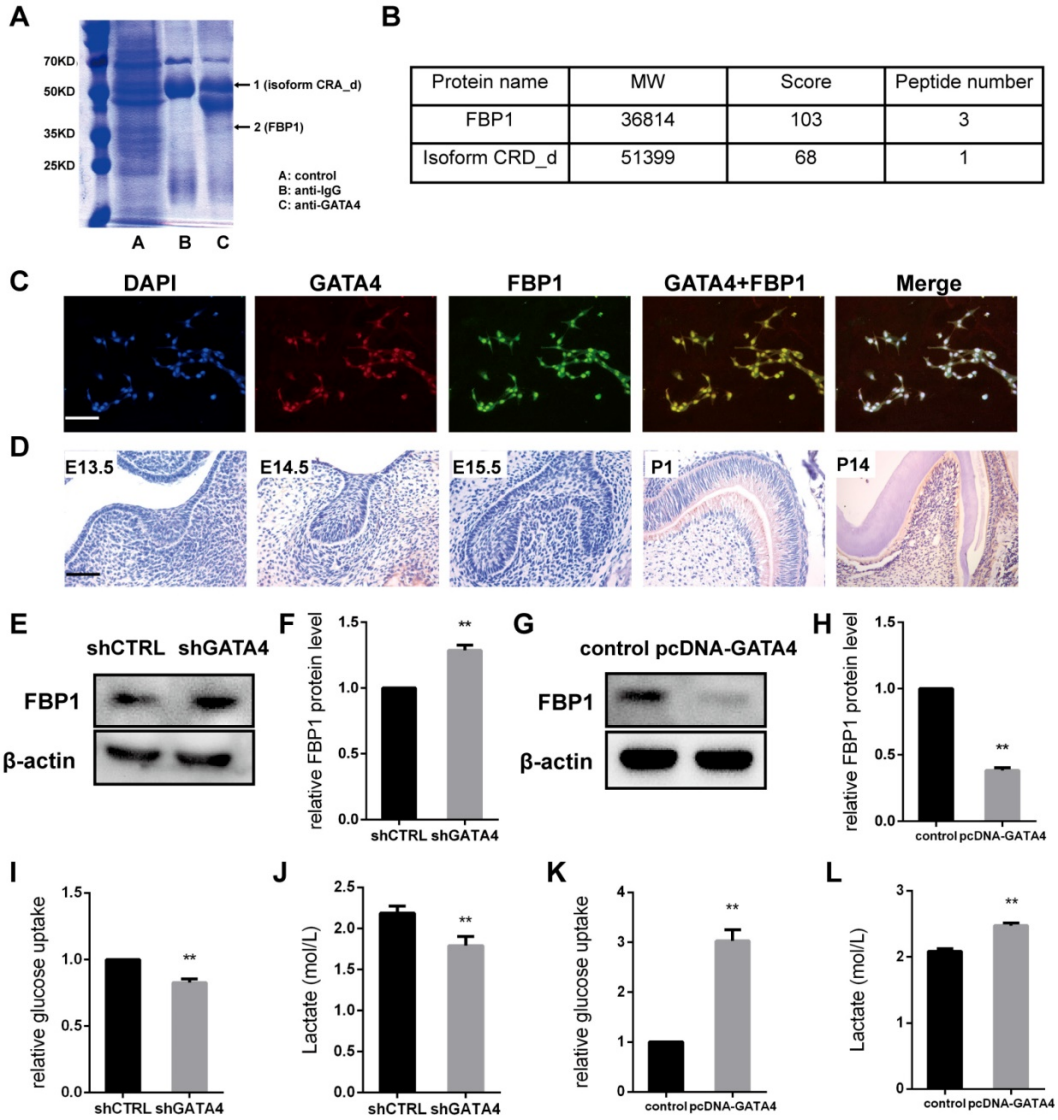
**GATA4 enhanced glycolysis by negatively regulating FBP1 in DPSCs.** (**A**) Co-immunoprecipitated proteins were then separated using SDS-PAGE and stained. (**B**) Mass spectrometry followed by peptide sequencing identified the two proteins as fructose-1,6-bisphosphatase 1 (FBP1) and isoform CRA_d. (**C**) Immunofluorescence staining revealed that GATA4 co-localizes with FBP1 in the nucleus in DPSCs (Bar: 100 μm). (**D**) Expression pattern of GATA4 during tooth development at embryonic day 13.5 (E13.5), E14.5, E15.5, P1, and P14 (Bar: 50 μm). (**E**) FBP1 expression was tested by western blotting after infection with GATA4 lentivirus in DPSCs. (**F**) Quantitative analysis of western blotting bands from (E). (**G**) FBP1 expression was tested by western blotting after GATA4 overexpression in DPSCs. (**H**) Quantitative analysis of western blotting bands from (G). (**I, J**) knockdown of GATA4 resulted in decreased glucose consumption and lactate production. (**K, L**) Overexpression of GATA4 resulted in increased glucose consumption and lactate production. Data expressed as the mean ± standard deviation; n = 3. ^**^P < 0.01.

To identify GATA4-interacting proteins, a schematic outline of the immunoprecipitation-mass spectrometry procedure used in this study is presented. Co-immunoprecipitated proteins were then separated using SDS-PAGE and stained (Fig. 6A). Coomassie staining of the gels loaded with GATA4-IP identified two bands, including one with a molecular mass of approximately 37 kDa. Mass spectrometry followed by peptide sequencing identified the 37-kDa protein as fructose-1,6-bisphosphatase 1 (FBP1) (Fig. 6B). Moreover, immunofluorescence staining revealed that GATA4 co-localizes with FBP1 to the nucleus (Fig. 6C). These results confirmed the interaction between GATA4 and FBP1.

In the present study, our results revealed that FBP1 is widely expressed in odontoblasts and dental pulp cells but is minimally visible in tooth germ at E13.5, E14.5, and E15.5 (Fig. 6D). Next, we knocked down GATA4 level using lentivirus-mediated infection with a specific shRNA and assessed FBP1 protein expression in DPSCs. FBP1 expression was markedly increased in the shGATA4 group (Fig. 6E, F). Moreover, western blotting assays also confirmed that overexpression of GATA4 reduced FBP1 protein level (Fig. 6G, H). Collectively, these data indicated that GATA4 regulated FBP1 expression in DPSCs.

### GATA4 enhanced proliferation, odonto/osteogenic differentiation and glycolysis by repressing FBP1 in DPSCs

**Figure 7:**
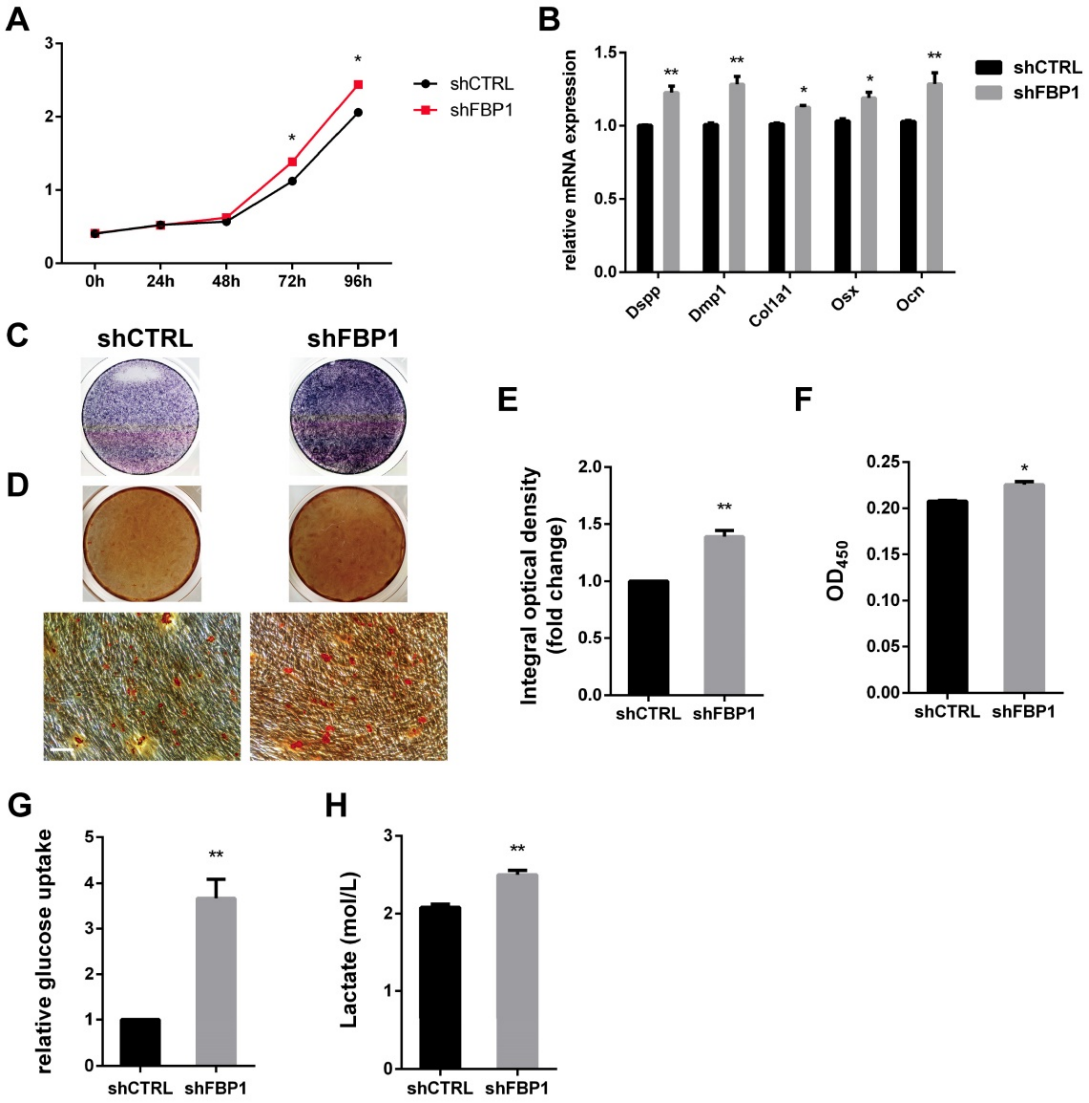
** Effect of FBP1 on proliferation, odonto/osteogenic differentiation and glycolysis of DPSCs.** (**A**) The CCK8 assay was used to analyse the proliferation of DPSCs after FBP1 knockdown. (**B**) Expressions of odonto/osteogenic markers (Dspp, Dmp1, Col1a1, Osx, and Ocn) were assessed by qRT-PCR. (**C**) ALP staining was observed after 7 days of mineralization. (**D**) ARS staining was performed after mineralization (Bar: 100 μm). (**E**) Quantitative assessment of ALP-positive areas after 7 days of osteogenic induction. (**F**) Semi-quantitative estimation of calcium. (**G, H**) Silent FBP1 expression significantly increased glucose utilization and lactate production. Data expressed as the mean ± standard deviation; n = 3. ^*^P < 0.05, ^**^P < 0.01

Stem cells mainly rely on glycolysis to support cellular events 31,32. As shown in (Fig. 6I, J), DPSCs in the shGATA4 group revealed that the knockdown of GATA4 resulted in decreased glucose consumption and lactate production. Consistently, the overexpression of GATA4 increased glucose consumption (Fig. 6K) and lactate production (Fig. 6L). These results indicate that GATA4 has a profound effect on glucose metabolism, especially glycolysis. Moreover, FBP1 is a key regulatory enzyme during the process of gluconeogenesis that suppresses glycolysis. Therefore, we assumed that GATA4 negatively regulated FBP1 expression in DPSCs. To further confirm whether FBP1 was associated with GATA4-mediated regulation of glucose metabolism, we used small hairpin RNA targeting FBP1 to reduce the expression of FBP1 in DPSCs. Silencing FBP1 expression significantly increased glucose utilization and lactate production (Fig. 7G, H). Furthermore, the proliferation rates of DPSCs were increased in response to knockdown of FBP1 expression using CCK8 assays (Fig. 7A). ALP staining revealed significant increase in ALP-positive areas in shFBP1 group compared with the control group (Fig. 7C, E). Moreover, the results of the alizarin red staining assay were consistent with the ALP assay (Fig. 7D, F). Moreover, the expression of odonto/osteogenic markers (Dspp, Dmp1, Col1a1, Osx, and Ocn) at the mRNA level was upregulated in the shFBP1 group (Fig. 7B). Collectively, GATA4 enhances proliferation, odonto/osteogenic differentiation and glycolysis through regulating FBP1 in DPSCs.

## Discussion

In the present study, we demonstrated that GATA4 is important for root formation and odontoblast polarity as it promotes growth, differentiation of dental mesenchymal cells around the root and affects glucose metabolism of DPSCs *via* negatively regulating FBP1.

GATA4 has been well studied, given its central role in cardiac development 4,5. Several studies have revealed that GATA4 is an important factor in osteoblasts 6,7. Moreover, our previous study showed that GATA4 is essential for root formation, as it promotes the proliferation of dental mesenchymal cells around the root 9. In this study, we found that GATA4 strongly located in a large number of dental pulp cells in mice during tooth development. *Wnt1-Cre; GATA4^fl/fl^* mice teeth also showed that root dentin thickness was significantly decreased. However, its role in postnatal odontoblast development and dentin formation has not been precisely studied.

Well-differentiated odontoblasts consistently exhibit a tall columnar shape, whereas H&E-stained sections of* Wnt1-Cre; GATA4^fl/fl^* mice teeth showed that the odontoblasts lose polarity and exhibit reduced height and flattened morphology. Thus, *Wnt1-Cre; GATA4^fl/fl^* mice odontoblasts are dysfunctional. Consistently, several markers of odontoblasts and dentin provide convincing evidence supporting a critical role for GATA4 in odontoblast differentiation and dentin formation. DSPP, an important regulator of dentin mineralization, is located within the pre-dentin and dentinal tubules. Col-1 was expressed in control odontoblasts and dentin. DCN is normally localized in the pre-dentin and pulp core 33. However, in *Wnt1-Cre; GATA4^fl/fl^* mutants, these molecules were downregulated. These findings suggest that ablation of GATA4 in the dental mesenchymal cells resulted in the impairment of tooth root by disturbing molecular expression. Furthermore, in the mutants, PCNA-labelled proliferating cells in the odontoblasts and DPCs were decreased at P14. There, we also built an animal model. Lentivirus that overexpresses GATA4 and the control were injected under the buccal periosteum of the left mandibular first molar. The results provide convincing evidence that GATA4 promote odontoblast differentiation and dentin formation.

Coincident with the *in vivo* results, the proliferation and differentiation of DPSCs were affected *in vitro* by inducing GATA4 knockdown. Interestingly, we found that GATA4 upregulates RUNX2, as determined by analysis of both mRNA expression and secretion of RUNX2 protein. This is consistent with our previous study 9 and some prior work from Khalid et al 34. However, there is some evidence indicates that GATA4 downregulates RUNX2. Song et al 35. Showed that GATA4 plays a negative role in osteoblastogenesis by down-regulating RUNX2. The apparent discrepancy observed in these two studies may arise because differences in cells and stages of cells. The regulation of GATA4 on RNUX2 was not fully understood, and will require further investigation.

Glycometabolic reprogramming plays an important role in stem cell differentiation. Glycometabolism was reprogrammed *via* augmentation of both mitochondrial OXPHOS and glycolysis. Wang et al*.* 11 reported that glycolysis increased when DPSCs initiate differentiation. In this study, the immunoprecipitation-mass spectrometry procedure was used to confirm the interaction between GATA4 and FBP1. FBP1 is a key regulatory enzyme during the process of gluconeogenesis 12 that is ubiquitously expressed in different tissues. Interestingly, our work showed that GATA4 knockdown in the DPSCs increased the expression of FBP1, while GATA4 overexpression in the DPSCs decreased FBP1 expression. Our results revealed that GATA4 could enhance proliferation, odonto/osteogenic differentiation and glycolysis in DPSCs. However, as a glycolysis antagonist, FBP1 deficiency increased glucose utilization, lactate production, and enhance proliferation, odonto/osteogenic differentiation in DPSCs. Therefore, GATA4 appears to regulate tooth root development through negatively regulating FBP1. However, the specific mechanism of FBP1 in tooth development needs further exploration.

Our work substantiates the involvement of both GATA4 and FBP1 in root development and dentin formation. The results therefore provide novel insights into the aetiology of root defects and open the possibility for further studies on signalling molecules that promote tooth development. From a clinical perspective, normal root development is critical for the function of dentition. GATA4 regulation in tooth root can potentially promote to increase the thickness of dentin or lengthen the root, thus stabilizing the dentition. Information generated from this study will lay the foundation for root regeneration, which can be used to support potential therapeutic approaches to restore dentition in order to provide a biologic solution for a biologic problem.

## Supplementary Material

Supplementary figures and tables.
