# Supplementary material for: GATA Binding Protein 4 Regulates Tooth Root Dentin Development via FBP1
Source: Int J Biol Sci. 2020 Jan 1;16(1):181–93. doi: 10.7150/ijbs.36567 (PMC6930368; doi:10.7150/ijbs.36567)
Supplement: Supplementary file 1 — Supplementary figures and tables. [file ijbsv16p0181s1.pdf]

Table S1

Name	Source	Identifiers
anti-GATA4	Abcam	#ab84593
anti-FBP1	Abcam	#ab109732
anti-DSPP	Santa Cruz	#sc-73632
anti-COL-1	Abcam	#ab21286
anti-DCN	BOSTER	#PB0132
anti-PCNA	BOSTER	#BM0104
anti-RUNX2	Abcam	#ab76956
anti-OSX	Abcam	#ab22552
anti-OPN	Abcam	#ab63856
anti-OCN	Abcam	#ab93876
anti-BMP4	Abcam	#ab39973
anti-β-actin	Servicebio	#GB13001-3
anti-GAPDH	Bioworld	#AP0063

The primary antibodies were listed as supplementary data (Table 1).
